# Sodium *N*-bromo-2-chloro­benzene­sulfonamidate sesquihydrate

**DOI:** 10.1107/S160053681102071X

**Published:** 2011-06-11

**Authors:** B. Thimme Gowda, Sabine Foro, K. Shakuntala

**Affiliations:** a Department of Chemistry, Mangalore University, Mangalagangotri 574 199, Mangalore, India; b Institute of Materials Science, Darmstadt University of Technology, Petersenstrasse 23, D-64287 Darmstadt, Germany

## Abstract

In the title compound, Na^+^·C_6_H_4_BrClNO_2_S^−^·1.5H_2_O, one water mol­ecule has crystallographically imposed twofold symmetry. The Na^+^ cation shows a pseudo-octa­hedral coordination provided by three O atoms of water mol­ecules and three sulfonyl O atoms of different *N*-bromo-2-chloro­benzene­sulfonamidate anions. The S—N distance of 1.579 (6) Å is consistent with an S=N double-bond character. The crystal structure is stabilized by O—H⋯Br, O—H⋯N and O—H⋯O hydrogen bonds.

## Related literature

For background to the chemistry of *N*-haloaryl­sulfonamides, see: Gowda & Shetty (2004 ▶); Usha & Gowda (2006 ▶). For our study of the effect of substituents on the structures of *N*-haloaryl­sulfonamides, see: Gowda, Kožíšek *et al.* (2007 ▶); Gowda, Usha *et al.* (2007 ▶). For related structures, see: George *et al.* (2000 ▶); Olmstead & Power (1986 ▶). For an isostructural compound, see: Gowda *et al.* (2010 ▶). 
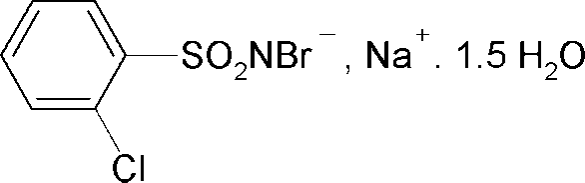


## Experimental

### 

#### Crystal data

Na^+^·C_6_H_4_BrClNO_2_S^−^·1.5H_2_O
*M* *_r_* = 319.53
Monoclinic, 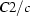

*a* = 11.200 (2) Å
*b* = 6.728 (1) Å
*c* = 28.304 (3) Å
β = 100.94 (1)°
*V* = 2094.0 (5) Å^3^
*Z* = 8
Mo *K*α radiation
μ = 4.41 mm^−1^
*T* = 293 K
0.34 × 0.30 × 0.14 mm

#### Data collection

Oxford Diffraction Xcalibur diffractometer with Sapphire CCD area detector
Absorption correction: multi-scan (*CrysAlis RED*; Oxford Diffraction, 2009 ▶) *T*_min_ = 0.316, *T*_max_ = 0.578
7442 measured reflections
2147 independent reflections
1955 reflections with *I* > 2σ(*I*)
*R*_int_ = 0.018

#### Refinement

*R*[*F* ^2^ > 2σ(*F* ^2^)] = 0.049
*wR*(*F* ^2^) = 0.128
*S* = 1.25
2147 reflections
141 parameters
4 restraints
H atoms treated by a mixture of independent and constrained refinement
Δρ_max_ = 2.27 e Å^−3^
Δρ_min_ = −1.19 e Å^−3^

### 

Data collection: *CrysAlis CCD* (Oxford Diffraction, 2009 ▶); cell refinement: *CrysAlis RED* (Oxford Diffraction, 2009 ▶); data reduction: *CrysAlis RED*; program(s) used to solve structure: *SHELXS97* (Sheldrick, 2008 ▶); program(s) used to refine structure: *SHELXL97* (Sheldrick, 2008 ▶); molecular graphics: *PLATON* (Spek, 2009 ▶); software used to prepare material for publication: *SHELXL97*.

## Supplementary Material

Crystal structure: contains datablock(s) I, global. DOI: 10.1107/S160053681102071X/rz2602sup1.cif

Structure factors: contains datablock(s) I. DOI: 10.1107/S160053681102071X/rz2602Isup2.hkl

Additional supplementary materials:  crystallographic information; 3D view; checkCIF report

## Figures and Tables

**Table 1:** Hydrogen-bond geometry (Å, °)

*D*—H⋯*A*	*D*—H	H⋯*A*	*D*⋯*A*	*D*—H⋯*A*
O3—H31⋯Br1^i^	0.82 (2)	2.70 (2)	3.518 (5)	171 (8)
O3—H32⋯N1	0.81 (2)	2.21 (5)	2.934 (7)	149 (8)
O3—H32⋯O2	0.81 (2)	2.51 (5)	3.232 (7)	148 (8)
O4—H41⋯N1^ii^	0.82 (2)	2.20 (3)	3.002 (7)	168 (8)

Symmetry codes: (i) 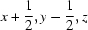; (ii) 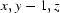.

**Fig. 1.**
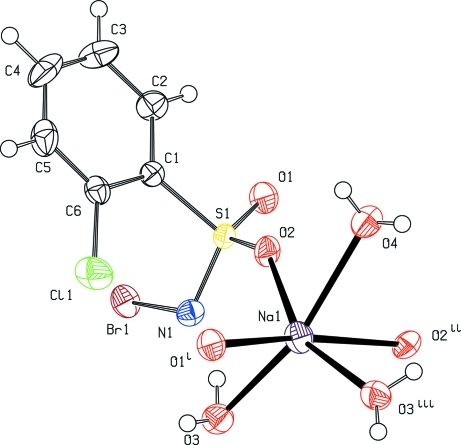
The molecular structure of the title compound, showing the asymmetric unit extended to show the coordination geometry for the Na+ ion. Displacement ellipsoids are drawn at the 50% probability level. H atoms are represented as small spheres of arbitrary radii (symmetry codes: (i) x + 1/2, y - 1/2, z; (ii) -x + 2, y, -z + 3/2; (iii) -x + 5/2, y - 1/2, -z + 3/2).

**Fig. 2.**
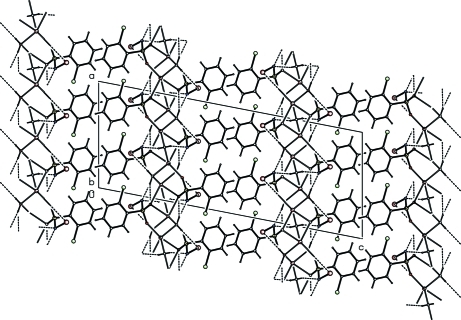
Crystal packing of the title compound with hydrogen bonding shown as dashed lines.

## supplementary crystallographic information

### Comment

The chemistry of N-halo arylsulfonamides are of interest in synthetic, mechanistic, analytical and biological chemistry (Gowda & Shetty, 2004; Usha & Gowda, 2006). In the present work, as a part of exploring the substituent effects on the crystal structures of N-haloarylsulfonamidates, the structure of sodium N-bromo-2-chlorobenzenesulfonamidate (I) has been determined (Fig. 1). The structure of (I) resembles those of sodium N-bromo-benzenesulfonamidate (II) (Gowda, Usha et al., 2007), sodium i>N-bromo-4-chlorobenzenesulfonamidate (III) (Gowda, Kožíšek et al., 2007) and other sodium N-chloro-arylsulfonamidates (George et al., 2000; Olmstead & Power, 1986), and is isostructural with the previously reported N-chloro-2-chloro-benzenesulfonamidate (Gowda et al., 2010) (IV).

In the title compound, one water molecule (O4) has crystallographically imposed twofold axis. The sodium ion shows octahedral coordination by three O atoms of water molecules and by three sulfonyl O atoms of three different N-bromo-2-chloro-benzenesulfonamide anions.

There is no interaction between the N and Na atoms in the molecule. The S—N distance of N1—S1, 1.579 (6)Å is consistent with a S—N double bond and is in agreement with the observed values of 1.578 (4)Å in (II), 1.588 (2) Å in (IV), and N1—S1, 1.574 (5)Å and N2—S2 1.579 (4)Å in (III).

The crystal packing consists of a two-dimensional polymeric layers running parallel to the ac plane (Fig. 2). The molecular packing is stabilized by O3—H31···Br1, O3—H32···N1, O3—H32···O2 and O4—H41···N1 hydrogen bonds (Table 1).

### Experimental

The title compound was prepared according to the literature method (Usha & Gowda, 2006). The purity of the compound was checked by determining its melting point. It was characterized by recording its infrared and NMR spectra. Prism like yellow single crystals of the title compound used in X-ray diffraction studies were obtained from slow evaporation of its aqueous solution at room temperature.

### Refinement

The H atoms bound to O3 were located in a difference Fourier map and later restrained to O—H = 0.82 (2) Å and H—H distance was restrained to 1.365 Å. The H atom bound to O4 was located in difference map and later restrained to O—H = 0.82 (2) Å. The other H atoms were positioned with idealized geometry using a riding model with C—H = 0.93 Å. All H atoms were refined with isotropic displacement parameters set to 1.2 times of the *U*_eq_ of the parent atoms. The residual electron-density features are located in the region of S1. The highest peak and the deepest hole are at 1.43 and 1.09 Å from S1, respectively.

### Crystal data

**Table:**

Na^+^·C_6_H_4_BrClNO_2_S^−^·1.5H_2_O	*F*(000) = 1256
*M**_r_* = 319.53	*D*_x_ = 2.027 Mg m^−^^3^
Monoclinic, *C*2/*c*	Mo *K*α radiation, λ = 0.71073 Å
Hall symbol: -C 2yc	Cell parameters from 4107 reflections
*a* = 11.200 (2) Å	θ = 2.9–27.8°
*b* = 6.728 (1) Å	µ = 4.41 mm^−^^1^
*c* = 28.304 (3) Å	*T* = 293 K
β = 100.94 (1)°	Prism, yellow
*V* = 2094.0 (5) Å^3^	0.34 × 0.30 × 0.14 mm
*Z* = 8	

### Data collection

**Table:**

Oxford Diffraction Xcalibur diffractometer with Sapphire CCD area detector	2147 independent reflections
Radiation source: fine-focus sealed tube	1955 reflections with *I* > 2σ(*I*)
graphite	*R*_int_ = 0.018
rotation method data acquisition using ω scans	θ_max_ = 26.4°, θ_min_ = 2.9°
Absorption correction: multi-scan (*CrysAlis RED*; Oxford Diffraction, 2009)	*h* = −13→13
*T*_min_ = 0.316, *T*_max_ = 0.578	*k* = −8→7
7442 measured reflections	*l* = −35→35

### Refinement

**Table:**

Refinement on *F*^2^	Primary atom site location: structure-invariant direct methods
Least-squares matrix: full	Secondary atom site location: difference Fourier map
*R*[*F*^2^ > 2σ(*F*^2^)] = 0.049	Hydrogen site location: inferred from neighbouring sites
*wR*(*F*^2^) = 0.128	H atoms treated by a mixture of independent and constrained refinement
*S* = 1.25	*w* = 1/[σ^2^(*F*_o_^2^) + (0.0182*P*)^2^ + 43.7119*P*] where *P* = (*F*_o_^2^ + 2*F*_c_^2^)/3
2147 reflections	(Δ/σ)_max_ = 0.001
141 parameters	Δρ_max_ = 2.27 e Å^−^^3^
4 restraints	Δρ_min_ = −1.19 e Å^−^^3^

### Special details

**Table:**

Experimental. CrysAlis RED (Oxford Diffraction, 2009) Empirical absorption correction using spherical harmonics, implemented in SCALE3 ABSPACK scaling algorithm.
Geometry. All e.s.d.'s (except the e.s.d. in the dihedral angle between two l.s. planes) are estimated using the full covariance matrix. The cell e.s.d.'s are taken into account individually in the estimation of e.s.d.'s in distances, angles and torsion angles; correlations between e.s.d.'s in cell parameters are only used when they are defined by crystal symmetry. An approximate (isotropic) treatment of cell e.s.d.'s is used for estimating e.s.d.'s involving l.s. planes.
Refinement. Refinement of *F*^2^ against ALL reflections. The weighted *R*-factor *wR* and goodness of fit *S* are based on *F*^2^, conventional *R*-factors *R* are based on *F*, with *F* set to zero for negative *F*^2^. The threshold expression of *F*^2^ > σ(*F*^2^) is used only for calculating *R*-factors(gt) *etc*. and is not relevant to the choice of reflections for refinement. *R*-factors based on *F*^2^ are statistically about twice as large as those based on *F*, and *R*-factors based on ALL data will be even larger.

### Fractional atomic coordinates and isotropic or equivalent isotropic displacement parameters (Å^2^)

**Table:**

	*x*	*y*	*z*	*U*_iso_*/*U*_eq_	
C1	0.8143 (5)	−0.3307 (9)	0.6083 (2)	0.0205 (12)	
C2	0.6903 (6)	−0.3509 (11)	0.5927 (2)	0.0311 (14)	
H2	0.6375	−0.3101	0.6125	0.037*	
C3	0.6437 (7)	−0.4309 (13)	0.5480 (3)	0.0433 (19)	
H3	0.5600	−0.4409	0.5377	0.052*	
C4	0.7209 (8)	−0.4957 (12)	0.5187 (3)	0.044 (2)	
H4	0.6892	−0.5508	0.4888	0.053*	
C5	0.8449 (8)	−0.4791 (11)	0.5336 (3)	0.0374 (17)	
H5	0.8971	−0.5243	0.5140	0.045*	
C6	0.8919 (6)	−0.3942 (9)	0.5782 (2)	0.0249 (13)	
Br1	0.88260 (6)	0.14784 (10)	0.62206 (2)	0.0312 (2)	
N1	0.9583 (5)	−0.0535 (8)	0.66407 (18)	0.0247 (11)	
Na1	1.1437 (2)	−0.5132 (4)	0.73529 (9)	0.0303 (6)	
O1	0.7549 (4)	−0.1730 (8)	0.68272 (16)	0.0324 (11)	
O2	0.9370 (4)	−0.3782 (7)	0.69654 (15)	0.0286 (10)	
O3	1.2055 (4)	−0.1890 (8)	0.70471 (17)	0.0335 (11)	
H31	1.246 (5)	−0.241 (12)	0.687 (2)	0.040*	
H32	1.1325 (19)	−0.199 (12)	0.695 (2)	0.040*	
O4	1.0000	−0.7830 (10)	0.7500	0.0336 (16)	
H41	0.979 (7)	−0.861 (9)	0.728 (2)	0.040*	
S1	0.86584 (13)	−0.2281 (2)	0.66713 (5)	0.0198 (3)	
Cl1	1.04842 (16)	−0.3824 (3)	0.59503 (7)	0.0414 (5)	

### Atomic displacement parameters (Å^2^)

**Table:**

	*U*^11^	*U*^22^	*U*^33^	*U*^12^	*U*^13^	*U*^23^
C1	0.023 (3)	0.017 (3)	0.021 (3)	0.001 (2)	0.003 (2)	0.002 (2)
C2	0.026 (3)	0.033 (4)	0.033 (3)	0.004 (3)	0.003 (3)	0.000 (3)
C3	0.030 (4)	0.050 (5)	0.045 (4)	−0.004 (4)	−0.007 (3)	−0.009 (4)
C4	0.056 (5)	0.043 (5)	0.027 (4)	−0.005 (4)	−0.009 (3)	−0.010 (3)
C5	0.055 (5)	0.032 (4)	0.028 (4)	−0.001 (3)	0.017 (3)	−0.005 (3)
C6	0.030 (3)	0.020 (3)	0.026 (3)	0.000 (3)	0.009 (3)	0.002 (2)
Br1	0.0358 (4)	0.0245 (3)	0.0342 (4)	0.0033 (3)	0.0092 (3)	0.0072 (3)
N1	0.022 (3)	0.025 (3)	0.025 (3)	0.003 (2)	0.001 (2)	0.002 (2)
Na1	0.0299 (14)	0.0312 (14)	0.0320 (14)	0.0047 (11)	0.0114 (11)	−0.0007 (11)
O1	0.030 (2)	0.040 (3)	0.030 (2)	0.001 (2)	0.0131 (19)	−0.006 (2)
O2	0.033 (2)	0.028 (2)	0.023 (2)	0.002 (2)	0.0021 (18)	0.0075 (19)
O3	0.027 (2)	0.039 (3)	0.034 (3)	0.002 (2)	0.006 (2)	−0.003 (2)
O4	0.044 (4)	0.024 (4)	0.030 (4)	0.000	0.000 (3)	0.000
S1	0.0209 (7)	0.0215 (7)	0.0170 (7)	0.0005 (6)	0.0038 (5)	0.0002 (6)
Cl1	0.0280 (8)	0.0478 (11)	0.0522 (11)	0.0018 (8)	0.0174 (8)	−0.0107 (9)

### Geometric parameters (Å, °)

**Table:**

C1—C2	1.382 (9)	Na1—O3^iii^	2.459 (5)
C1—C6	1.393 (8)	Na1—O3	2.493 (6)
C1—S1	1.793 (6)	Na1—O4	2.512 (6)
C2—C3	1.383 (10)	Na1—O2	2.534 (5)
C2—H2	0.9300	Na1—S1^ii^	3.381 (3)
C3—C4	1.378 (12)	Na1—H32	2.40 (9)
C3—H3	0.9300	O1—S1	1.444 (5)
C4—C5	1.378 (11)	O1—Na1^iv^	2.371 (5)
C4—H4	0.9300	O2—S1	1.448 (5)
C5—C6	1.396 (9)	O2—Na1^ii^	2.455 (5)
C5—H5	0.9300	O3—Na1^v^	2.459 (5)
C6—Cl1	1.729 (7)	O3—H31	0.82 (2)
Br1—N1	1.893 (5)	O3—H32	0.81 (2)
N1—S1	1.579 (6)	O4—Na1^ii^	2.512 (6)
Na1—O1^i^	2.371 (5)	O4—H41	0.82 (2)
Na1—O2^ii^	2.455 (5)	S1—Na1^ii^	3.381 (3)
			
C2—C1—C6	118.6 (6)	O2^ii^—Na1—S1^ii^	22.28 (11)
C2—C1—S1	117.5 (5)	O3^iii^—Na1—S1^ii^	80.37 (14)
C6—C1—S1	123.9 (5)	O3—Na1—S1^ii^	80.85 (13)
C1—C2—C3	120.9 (6)	O4—Na1—S1^ii^	98.86 (11)
C1—C2—H2	119.6	O2—Na1—S1^ii^	88.99 (13)
C3—C2—H2	119.6	O1^i^—Na1—H32	94.9 (14)
C4—C3—C2	120.2 (7)	O2^ii^—Na1—H32	93.2 (15)
C4—C3—H3	119.9	O3^iii^—Na1—H32	136.1 (7)
C2—C3—H3	119.9	O3—Na1—H32	19.0 (5)
C5—C4—C3	120.1 (7)	O4—Na1—H32	137.7 (5)
C5—C4—H4	120.0	O2—Na1—H32	61.1 (5)
C3—C4—H4	120.0	S1^ii^—Na1—H32	83.3 (15)
C4—C5—C6	119.7 (7)	S1—O1—Na1^iv^	153.3 (3)
C4—C5—H5	120.2	S1—O2—Na1^ii^	117.7 (3)
C6—C5—H5	120.2	S1—O2—Na1	149.0 (3)
C1—C6—C5	120.5 (6)	Na1^ii^—O2—Na1	88.25 (17)
C1—C6—Cl1	122.4 (5)	Na1^v^—O3—Na1	112.4 (2)
C5—C6—Cl1	117.1 (5)	Na1^v^—O3—H31	104 (5)
S1—N1—Br1	110.3 (3)	Na1—O3—H31	94 (6)
O1^i^—Na1—O2^ii^	167.5 (2)	Na1^v^—O3—H32	142 (5)
O1^i^—Na1—O3^iii^	80.89 (18)	Na1—O3—H32	74 (6)
O2^ii^—Na1—O3^iii^	86.70 (18)	H31—O3—H32	112 (4)
O1^i^—Na1—O3	88.03 (19)	Na1—O4—Na1^ii^	87.5 (3)
O2^ii^—Na1—O3	96.71 (18)	Na1—O4—H41	116 (6)
O3^iii^—Na1—O3	117.45 (15)	Na1^ii^—O4—H41	119 (6)
O1^i^—Na1—O4	101.87 (19)	O1—S1—O2	114.5 (3)
O2^ii^—Na1—O4	78.11 (16)	O1—S1—N1	115.9 (3)
O3^iii^—Na1—O4	85.18 (16)	O2—S1—N1	104.8 (3)
O3—Na1—O4	156.70 (19)	O1—S1—C1	103.8 (3)
O1^i^—Na1—O2	115.86 (19)	O2—S1—C1	108.1 (3)
O2^ii^—Na1—O2	76.42 (19)	N1—S1—C1	109.5 (3)
O3^iii^—Na1—O2	157.31 (19)	O1—S1—Na1^ii^	74.5 (2)
O3—Na1—O2	80.02 (17)	N1—S1—Na1^ii^	126.1 (2)
O4—Na1—O2	76.68 (15)	C1—S1—Na1^ii^	119.0 (2)
O1^i^—Na1—S1^ii^	150.64 (16)		
			
C6—C1—C2—C3	−0.5 (10)	O1^i^—Na1—O4—Na1^ii^	−152.81 (18)
S1—C1—C2—C3	−179.3 (6)	O2^ii^—Na1—O4—Na1^ii^	39.93 (12)
C1—C2—C3—C4	1.4 (12)	O3^iii^—Na1—O4—Na1^ii^	127.57 (16)
C2—C3—C4—C5	−0.7 (13)	O3—Na1—O4—Na1^ii^	−39.3 (4)
C3—C4—C5—C6	−0.8 (12)	O2—Na1—O4—Na1^ii^	−38.72 (11)
C2—C1—C6—C5	−1.0 (10)	S1^ii^—Na1—O4—Na1^ii^	48.11 (6)
S1—C1—C6—C5	177.7 (5)	Na1^iv^—O1—S1—O2	73.2 (8)
C2—C1—C6—Cl1	−178.1 (5)	Na1^iv^—O1—S1—N1	−49.0 (8)
S1—C1—C6—Cl1	0.6 (8)	Na1^iv^—O1—S1—C1	−169.1 (7)
C4—C5—C6—C1	1.6 (11)	Na1^iv^—O1—S1—Na1^ii^	74.1 (7)
C4—C5—C6—Cl1	178.9 (6)	Na1^ii^—O2—S1—O1	1.2 (4)
O1^i^—Na1—O2—S1	−74.9 (6)	Na1—O2—S1—O1	−142.6 (5)
O2^ii^—Na1—O2—S1	107.4 (5)	Na1^ii^—O2—S1—N1	129.4 (3)
O3^iii^—Na1—O2—S1	150.4 (5)	Na1—O2—S1—N1	−14.4 (6)
O3—Na1—O2—S1	8.0 (6)	Na1^ii^—O2—S1—C1	−113.9 (3)
O4—Na1—O2—S1	−171.8 (6)	Na1—O2—S1—C1	102.3 (6)
S1^ii^—Na1—O2—S1	88.9 (6)	Na1—O2—S1—Na1^ii^	−143.8 (7)
O1^i^—Na1—O2—Na1^ii^	136.66 (17)	Br1—N1—S1—O1	−57.6 (4)
O2^ii^—Na1—O2—Na1^ii^	−41.0 (2)	Br1—N1—S1—O2	175.1 (3)
O3^iii^—Na1—O2—Na1^ii^	2.0 (6)	Br1—N1—S1—C1	59.4 (4)
O3—Na1—O2—Na1^ii^	−140.47 (18)	Br1—N1—S1—Na1^ii^	−146.92 (16)
O4—Na1—O2—Na1^ii^	39.77 (14)	C2—C1—S1—O1	−5.0 (6)
S1^ii^—Na1—O2—Na1^ii^	−59.58 (15)	C6—C1—S1—O1	176.3 (5)
O1^i^—Na1—O3—Na1^v^	−108.3 (2)	C2—C1—S1—O2	117.0 (5)
O2^ii^—Na1—O3—Na1^v^	60.1 (2)	C6—C1—S1—O2	−61.7 (6)
O3^iii^—Na1—O3—Na1^v^	−29.7 (2)	C2—C1—S1—N1	−129.4 (5)
O4—Na1—O3—Na1^v^	135.6 (4)	C6—C1—S1—N1	51.9 (6)
O2—Na1—O3—Na1^v^	135.0 (2)	C2—C1—S1—Na1^ii^	74.7 (5)
S1^ii^—Na1—O3—Na1^v^	44.40 (17)	C6—C1—S1—Na1^ii^	−103.9 (5)

Symmetry codes: (i) *x*+1/2, *y*−1/2, *z*; (ii) −*x*+2, *y*, −*z*+3/2; (iii) −*x*+5/2, *y*−1/2, −*z*+3/2; (iv) *x*−1/2, *y*+1/2, *z*; (v) −*x*+5/2, *y*+1/2, −*z*+3/2.

### Hydrogen-bond geometry (Å, °)

**Table:**

*D*—H···*A*	*D*—H	H···*A*	*D*···*A*	*D*—H···*A*
O3—H31···Br1^i^	0.82 (2)	2.70 (2)	3.518 (5)	171 (8)
O3—H32···N1	0.81 (2)	2.21 (5)	2.934 (7)	149 (8)
O3—H32···O2	0.81 (2)	2.51 (5)	3.232 (7)	148 (8)
O4—H41···N1^vi^	0.82 (2)	2.20 (3)	3.002 (7)	168 (8)

Symmetry codes: (i) *x*+1/2, *y*−1/2, *z*; (vi) *x*, *y*−1, *z*.
